# Modulation of the Oncogenic LINE-1 Regulatory Network in Non-Small Cell Lung Cancer by Exosomal miRNAs

**DOI:** 10.3390/ijms251910674

**Published:** 2024-10-03

**Authors:** Abeer A. I. Hassanin, Kenneth S. Ramos

**Affiliations:** 1 Center for Genomic and Precision Medicine, Texas Medical Center, Texas A&M Institute of Biosciences and Technology, Houston, TX 77030, USA; ahassanin@tamu.edu; 2 Department of Animal Wealth Development, Faculty of Veterinary Medicine, Suez Canal University, Ismailia 41522, Egypt

**Keywords:** plasma exosomes, LINE-1, microRNA profiles, oncogenic signaling, non-small cell lung cancer

## Abstract

Several microRNAs (miRNAs), including miR-221-5p, Let-7b-5p, miR-21-5p, miR-9-5p, miR-126-3p, and miR-222-3p, were recently found to be enriched in circulating exosomes of patients with non-small cell lung cancers (NSCLCs). These miRNAs distinguished cancer cases from controls with high precision and were predicted to modulate the expression of genes within the oncogenic LINE-1 regulatory network. To test this hypothesis, plasma exosomes from controls, early, and late-stage NSCLC patients were co-cultured with non-tumorigenic lung epithelial cells for 72 h and processed for measurements of gene expression. Exosomes from late-stage NSCLC patients markedly increased the mRNA levels of LINE-1 ORF1 and ORF2, as well as the levels of target miRNAs in naïve recipient cells compared to saline or control exosomes. Late-stage exosomes also modulated the expression of oncogenic targets within the LINE-1 regulatory network, namely, ICAM1, AGL, RGS3, RGS13, VCAM1, and TGFβ1. In sharp contrast, exosomes from controls or early-stage NSCLC patients inhibited LINE-1 expression, along with many of the genetic targets within the LINE-1 regulatory network. Thus, late-stage NSCLC exosomes activate LINE-1 and miRNA-regulated oncogenic signaling in non-tumorigenic, recipient lung bronchial epithelial cells. These findings raise important questions regarding lung cancer progression and metastasis and open the door for the exploration of new therapeutic interventions.

## 1. Introduction

Lung cancer continues to be the primary cause of cancer-related mortality in the United States, accounting for approximately 1.8 million deaths in 2022 [1]. Non-small cell lung cancers (NSCLCs) account for more than 85% of all lung cancer cases and are classified by histological subtype into squamous cell lung cancers (SQCLCs), lung adenocarcinomas (LUADs), adeno-squamous carcinomas, and large cell carcinomas [2,3,4]. Metastasis is a significant contributor to mortality in patients with lung cancer, where exosomes and their cargos, together with microenvironmental influences, play crucial roles in facilitating tumor migration and invasion [5,6]. Recent evidence indicates that exosomes transport physiologically active components to distant sites and organs, therefore guiding metastasis by creating metastatic pre-niches and promoting tumor development [7,8]. Cancer cells release considerably larger numbers of exosomes than normal cells, with tumor-derived exosomes transferring their RNA, miRNA, and protein cargos to recipient cells to modulate cellular functions [6,9,10,11]. MicroRNAs are regarded as the most biologically active molecules due to their gene regulatory functions [12].

MicroRNAs are small noncoding RNAs ranging in length from 17 to 24 nucleotides that mediate post-transcriptional gene silencing by binding to the 3′ end of the target mRNA untranslated region (UTR) or to the open reading frame (ORF) region [13]. The roles of miRNAs in cell proliferation, differentiation, migration, disease initiation, and progression are among the most extensively studied biological processes. MicroRNAs are frequently integrated into exosomes as signaling elements that play crucial functions in regulatory control [14,15]. Numerous cancers including lung cancer have been linked to altered profiles of miRNA expression, and exosomal miRNAs have been proposed as noninvasive biomarkers for cancer diagnosis [16]. Due to their role in carcinogenesis, cancer prognosis, and response to treatment, exosome-derived miRNAs may be more suitable than mRNAs or proteins as cancer biomarkers [7].

A close link between LINE-1 and miRNAs was established in studies that identified miRNAs that share sequence homology with retrotransposons [17], or that contain retrotransposon-related hairpin sequences [18]. Several human miRNAs originate from LINE-1, Alu, or MIR elements [19], and 85% of all known miRNA target sites within the genome overlap with LINE-1 and Alu elements [18]. However, little is known about how LINE-1 interacts with functional noncoding RNAs in NSCLC. Therefore, the main aim of the present study was to explore genetic interactions between genes within the LINE-1 regulatory network and eight exosomal miRNAs in lung cancer patients. We also examined the impact of these functional interactions in modulating oncogenic signaling pathways in naïve recipient lung epithelial cells. Evidence is presented here that late-stage NSCLC exosomes activate miRNA-regulated oncogenic signaling in non-tumorigenic, recipient lung bronchial epithelial cells.

## 2. Results

### 2.1. Exosome Characterization

Concentrations and diameters of exosomes for each group are displayed in Supplementary Figure S1(A1–A3). The exosomal identity of all preparations was confirmed by Western blotting detection of exosome protein markers including Alix, Flotillin-1, and CD-9 (Supplementary Figure S1B).

### 2.2. Predicted Interactions between Genes within the LINE-1 Regulatory Network and Exosomal miRNAs

**Figure 1:**
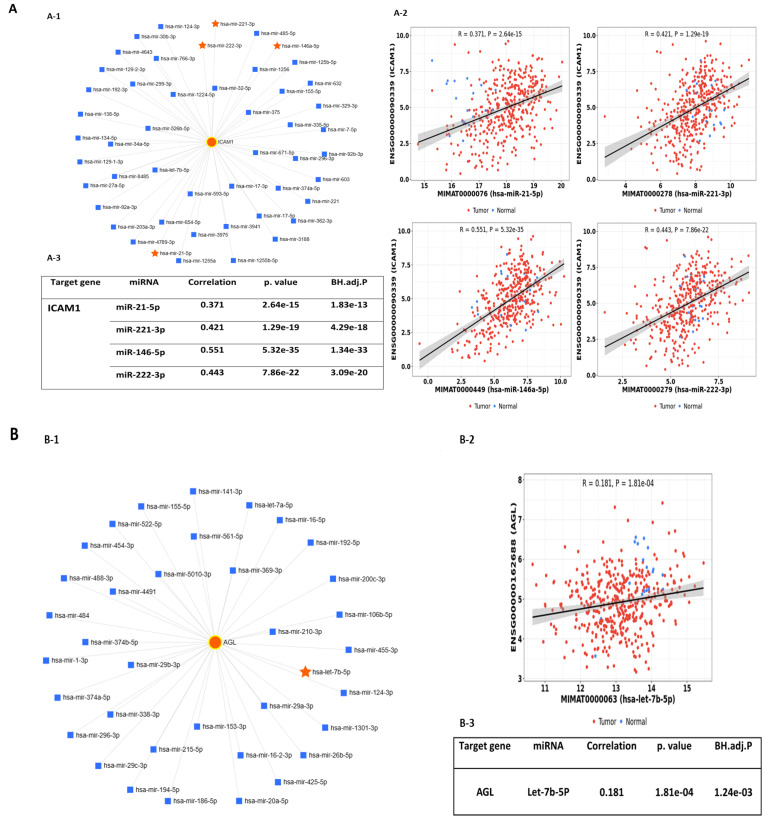

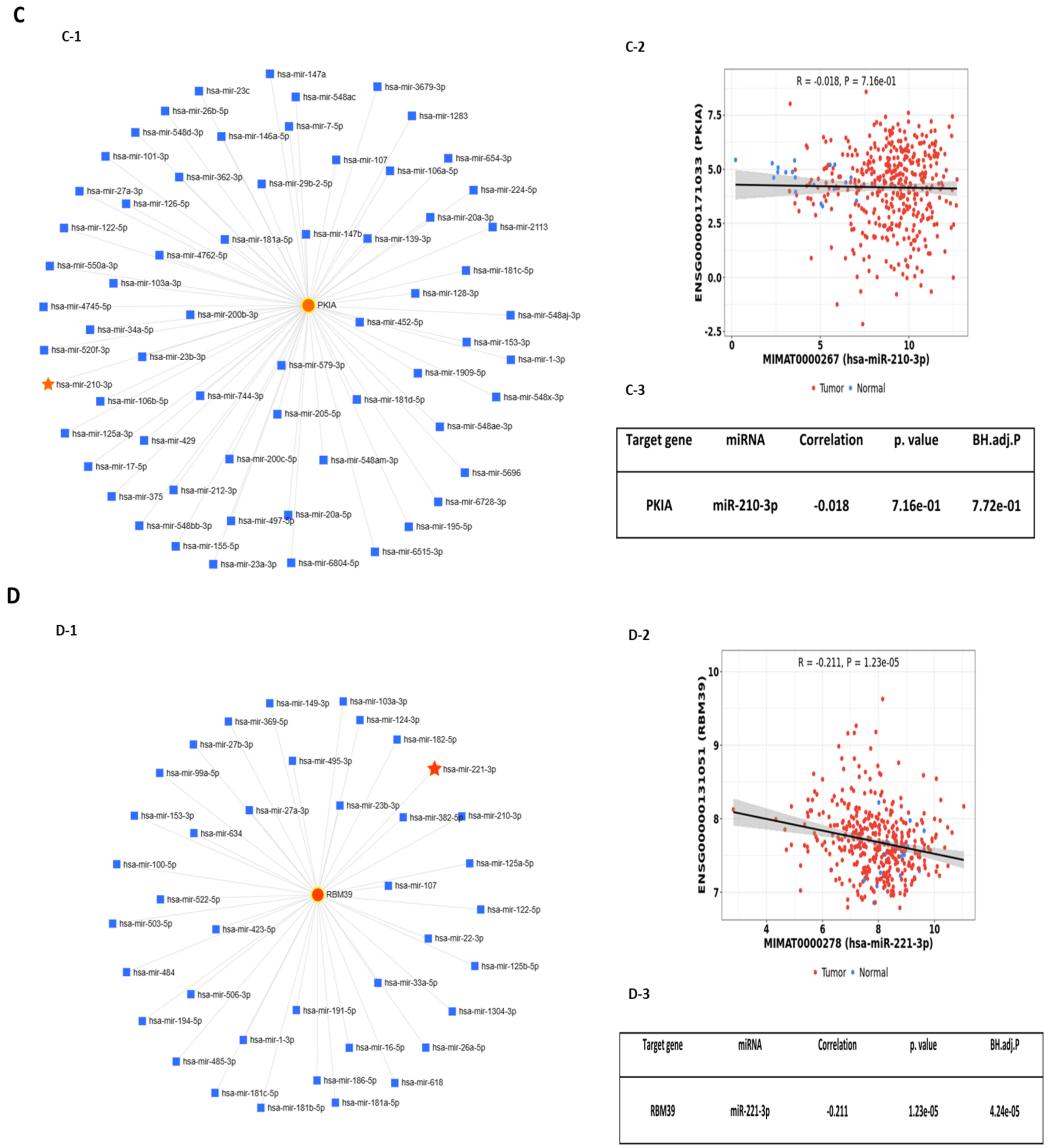

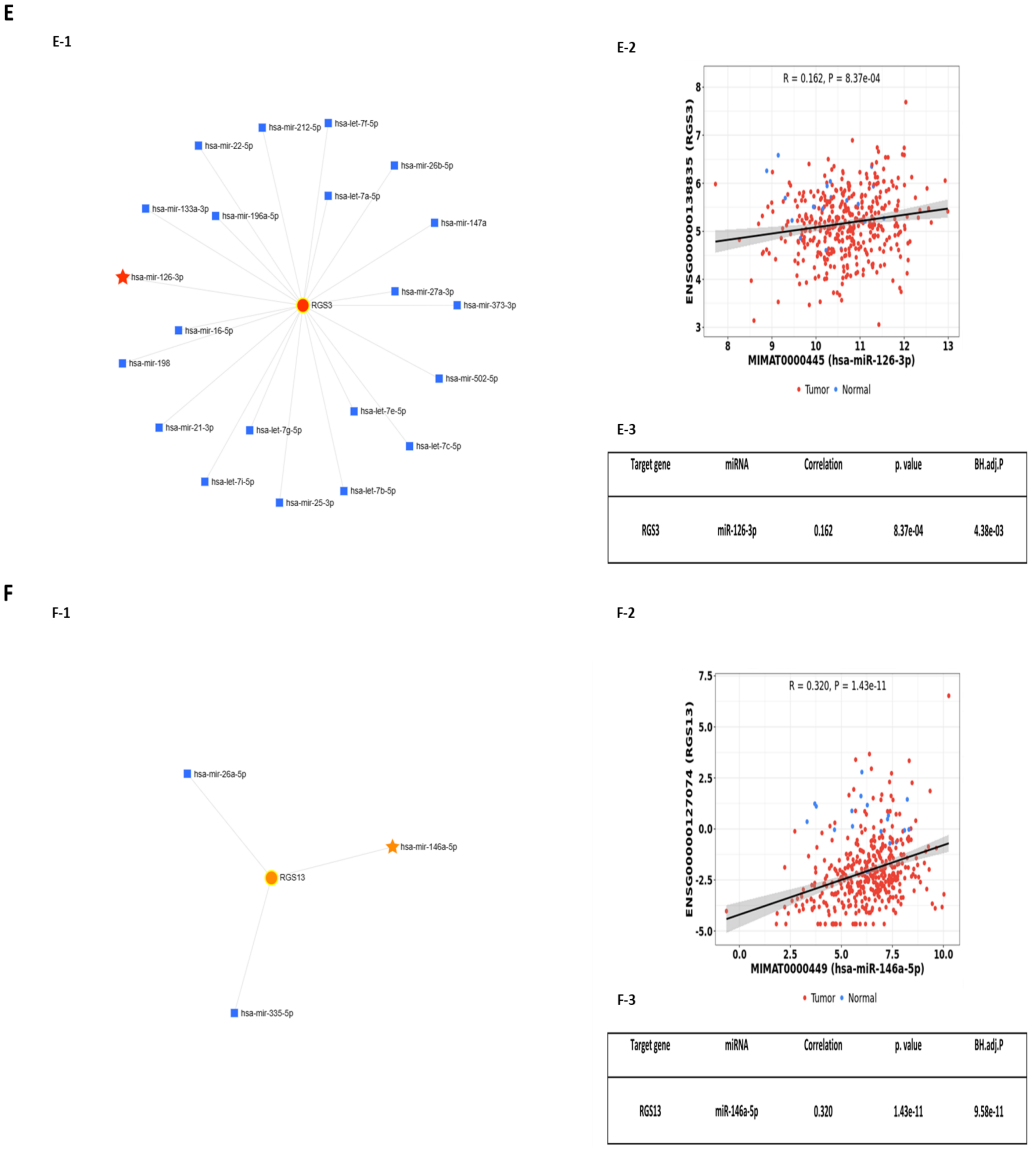

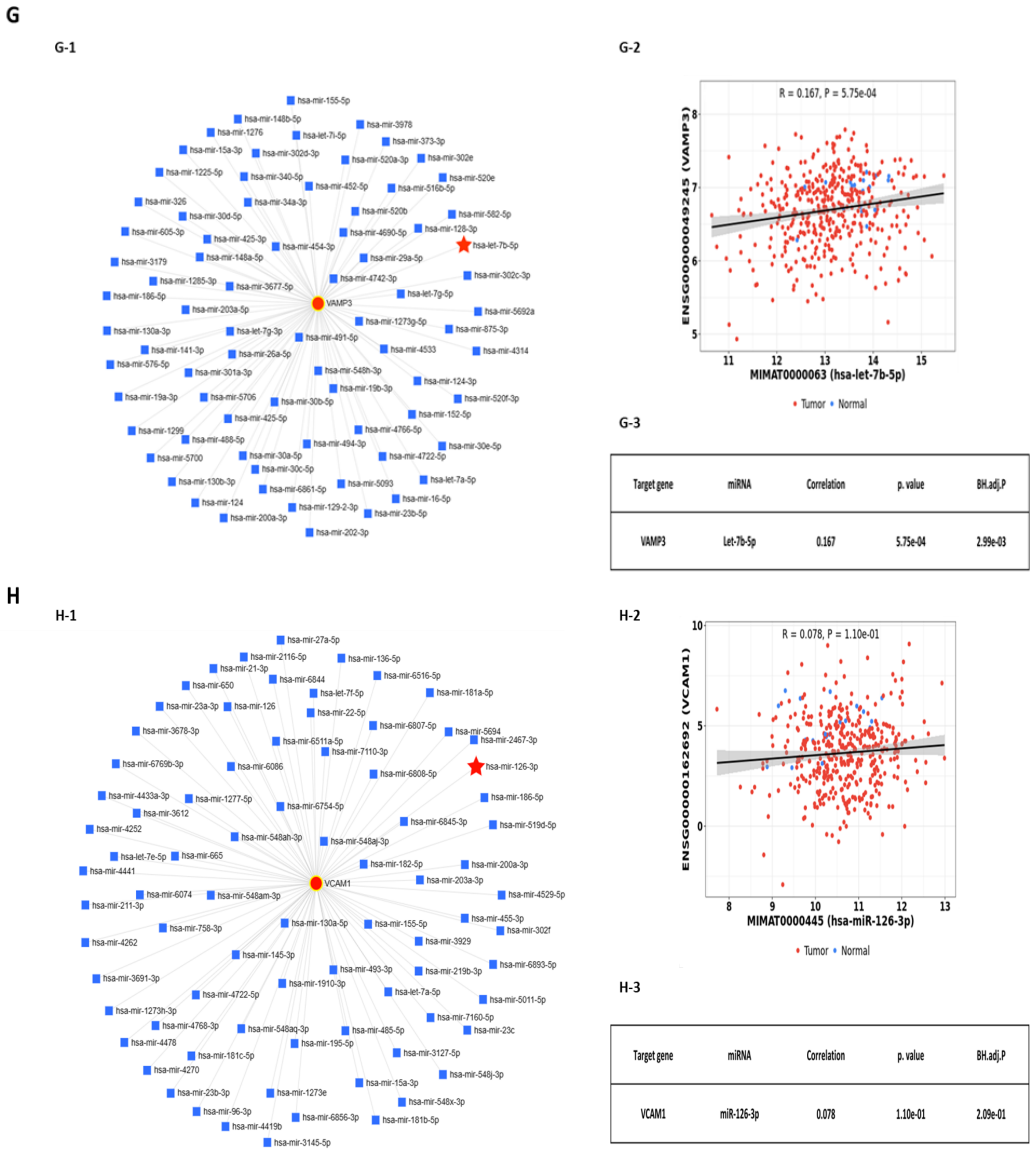

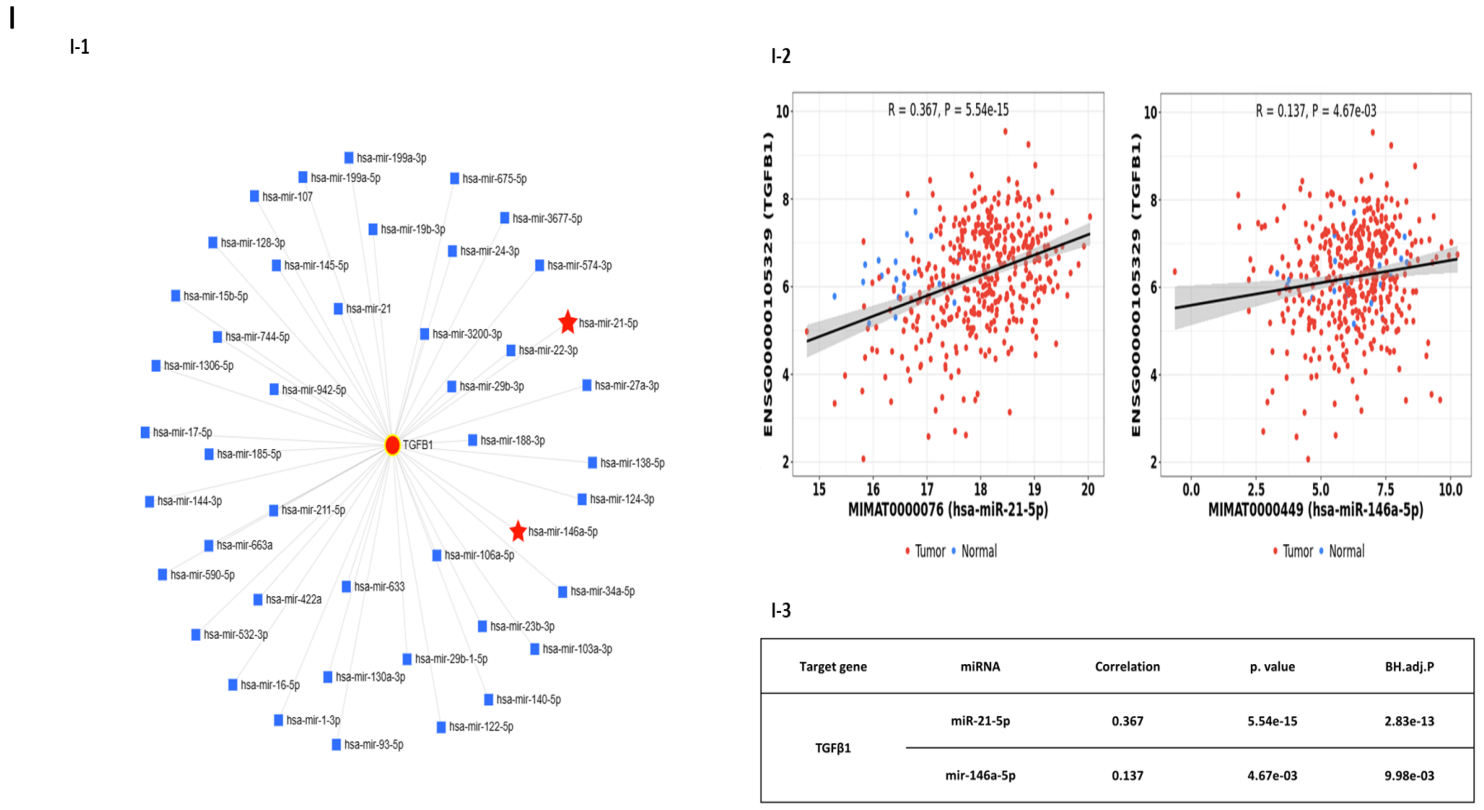
(**A-1**–**I-1**) Predicted genes within the LINE-1 regulatory network identified as validated targets of plasma exosomal miRNAs and their correlation scatter plots. (**A-1**) ICAM1 was a validated target for miR-21-5p, miR-146a-5p, miR-221-3p, and miR-222-3p ; (**B-1**) AGL was a validated target for let-7b-5p; (**C-1**) PKIA was a validated target for miR-210-3p ; (**D-1**) RBM39 was a validated target for miR-221-3p; (**E-1**) RGS3 was a validated target for miR-126-3p ; (**F-1**) RGS13 was a validated target for miR-146a-5p; (**G-1**) VAMP3 was a validated target for Let-7b-5p; (**H-1**) VCAM1was a validated factor for miR-126-3p; (**I-1**) TGFβ1 was a validated target for miR-21-5p and miR-146a-5p. Orange circles identify LINE-1 network genes, orange stars identify exosomal miRs, and blue squares identify regulatory miRNAs. (**A-2–I-2**) Scatter plots with Pearson correlation coefficients for the selected miRNAs and their predicted target genes (two-tailed test of significance (0.05 level). (**A-3–I-3**) Tables summarizing Pearson correlation coefficients of miRNAs and their predicted target genes.

**Figure 2:**
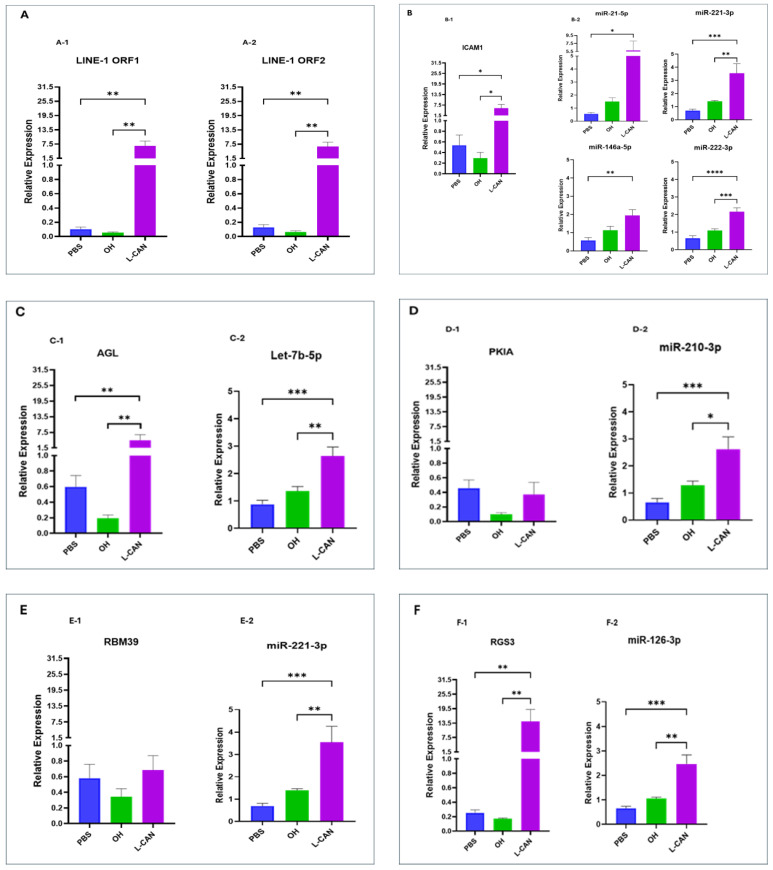

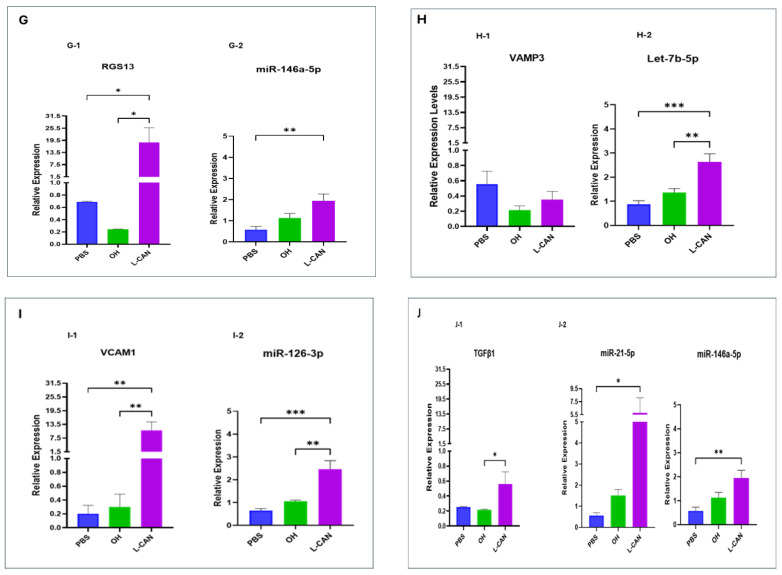
mRNA levels of LINE-1 and genes within its regulatory network along with their correlated miRNAs molecules in naïve BEAS-2B cells co-cultured with late-stage NSCLC plasma exosomes. (**A**) Expression of LINE-1 ORF1 (**A-1**) and ORF2 mRNAs (**A-2**). (**B**) Expression of ICAM1 mRNA (**B-1**) and four positively correlated miRNAs molecules (miR-21-5p, miR-221-3p, miR-146a-5p, and miR-222-3p) (**B-2**). (**C**) Expression of AGL (**C-1**) and Let-7b-5p (**C-2**). (**D**) Inverse correlation between PKIA mRNA (**D-1**) and miR-210-3p (**D-2**). (**E**) RBM39 gene (**E-1**) and miR-221-3p (**E-2**). (**F**) Expression of RGS3 (**F-1**) and miR-126-3p (**F-2**). (**G**) Expression of RGS13 (**G-1**) and miR-146a-5p (**G-2**). (**H**) Negative correlation between VAMP3 (**H-1**) and Let-7b-5p (**H-2**). (**I**) VCAM1 gene expression (**I-1**) and miR-126-3p (**I-2**). (**J**) TGFβ1 expression (**J-1**) and miR-21-5p and miR-146a-5p (**J-2**). PBS: BEAS-2B cells co-cultured with PBS for 72h, OH: BEAS-2B cells co-cultured with plasma exosomes from ostensibly healthy individuals for 72h, L-CAN: BEAS-2B cell co-cultured with plasma exosomes from late-stage NSCLC patients for 72h. *n* = 3 independent experiments and six replicates per sample. * *p* < 0.05, ** *p* < 0.005, *** *p* < 0.001, **** *p* < 0.0001.

**Figure 3:**
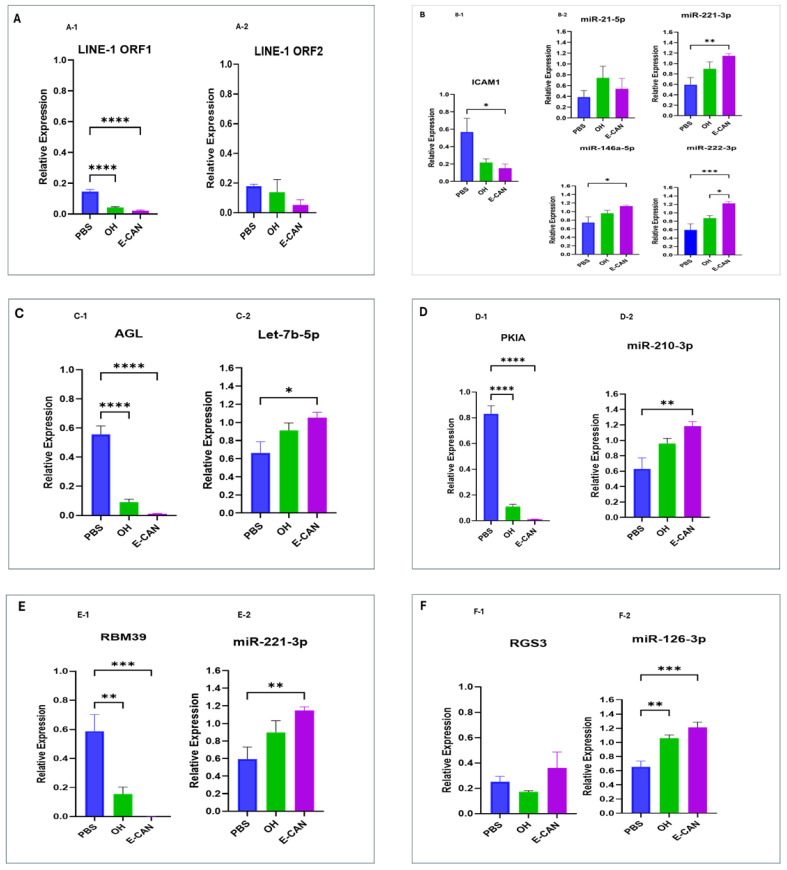

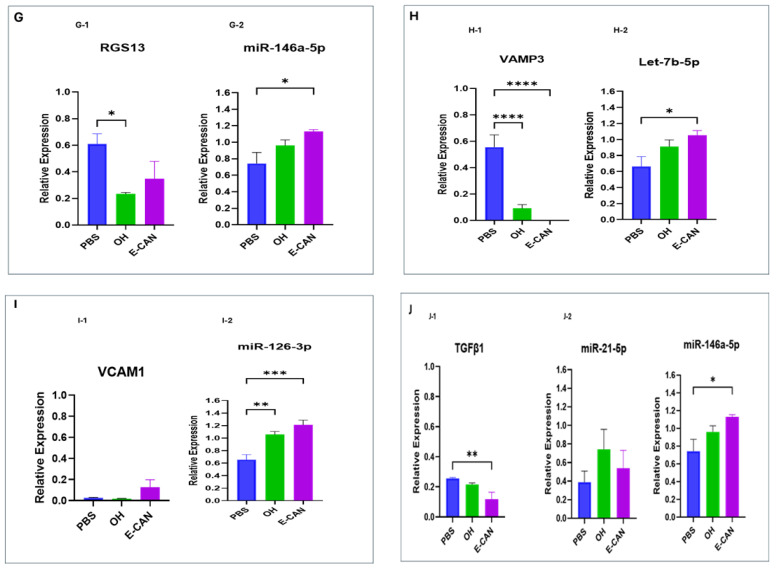
mRNA levels of LINE-1 and genes within its regulatory network along with their correlated miRNAs molecules in naïve BEAS-2B cell co-cultured with early-stage NSCLC plasma exosomes. (**A**) Expression of both LINE-1 ORF1 (**A-1**) and ORF2 mRNAs (**A-2**). (**B**) Expression of ICAM1 mRNA (**B-1**) and four positively correlated miRNAs molecules (miR-21-5p, miR-221-3p, miR-146a-5p, and miR-222-3p) (**B-2**). (**C**) Expression of AGL (**C-1**) and Let-7b-5p (**C-2**). (**D**) Downregulated expression of the PKIA gene (**D-1**) and activation by miR-210-3p (**D-2**). (**E**) Expression of RBM39 gene (**E-1**) and miR-221-3p miRNA (**E-2**). (**F**) Expression of RGS3 (**F-1**) and miR-126-3p (**F-2**). (**G**) Expression of RGS13 (**G-1**) and miR-146a-5p (**G-2**). (**H**) Expression of VAMP3 (**H-1**) and Let-7b miRNA (**H-2**). (**I**) Expression of VCAM1 (**I-1**) and miR-126-3p miRNA (**I-2**). (**J**) TGFβ1 expression (**J-1**) and miRNAs miR-21-5p and miR-146a-5p (**J-2**). PBS: BEAS-2B cells co-cultured with PBS for 72h to measure basal gene expression, OH: BEAS-2B cells co-cultured with plasma exosomes from ostensibly healthy individuals for 72 h, E-CAN: BEAS-2B cells co-cultured with plasma exosomes from early-stage NSCLC patients for 72 h. *n* = 3 independent experiments and six replicates per sample. * *p* < 0.05, ** *p* < 0.005, *** *p* < 0.001, **** *p* < 0.0001.

The miRNet2.0 database identified nine genes within the LINE-1 regulatory network as validated targets of exosomal miRNAs. These targets included Intercellular Adhesion Molecule 1 (ICAM1) (Figure 1A(A-1)), Amylo-1,6-glucosidase (AGL) (Figure 1B(B-1)), Protein kinase inhibitor alpha (PKIA) (Figure 1C(C-1)), RNA binding motif protein 39 (RBM39) (Figure 1D(D-1)), Regulators of G-protein signaling 3 (RGS3) (Figure 1E(E-1)), Regulators of G-protein signaling 13 (RGS13) (Figure 1F(F-1)), Vesicle-associated membrane protein 3 (VAMP3) (Figure 1G(G-1)), Vascular cell adhesion molecule1 (VCAM1) (Figure 1H(H-1)), and transforming growth factor-β1 (TGFβ1) (Figure 1I(I-1)). Pearson correlations for miRNAs and these genetic targets showed that ICAM1 positively correlated with miR-21-5p, miR-221-3p, miR-146-5p, and miR-222-3p (Figure 1A(A-2,A-3)) and that AGL positively correlated with Let-7b-5p (Figure 1B(B-2,B-3)). PKIA inversely correlated with miR-210-3p (Figure 1C(C-2,C-3)) and RBM39 inversely correlated with miR-221-3p (Figure 1D(D-2,D-3)). Both RGS3 (Figure 1E(E-2,E-3)) and VCAM1 (Figure 1H(H-2,H-3)) positively correlated with miR-126-3p, RGS13 positively correlated with mir-146a-5p (Figure 1F(F-2,F-3)), VAMP3 positively correlated with Let-7b-5p miRNA (Figure 1G(G-2,G-3)) and TGFβ1 positively correlated with miR-21-5p and mir-146a-5p (Figure 1I(I-2,I-3)). Thus, functional interactions may exist between exosomal miRNAs and genes within the LINE-1 genetic regulatory network that influence oncogenic signaling in NSCLC.

### 2.3. miRNA-Regulated Oncogenic Signaling in Human Lung Bronchial Epithelial Cells

To determine if predicted relationships between exosomal miRNAs and genes within the LINE-1 regulatory network translated into relevant functional interactions, plasma exosomes from OH subjects or early- and late-stage NSCLC patients matched for demographic characteristics were co-cultured with the non-tumorigenic BEAS-2B lung bronchial epithelial cell line for 72 h. BEAS-2B cells co-cultured with L-CAN exosomes exhibited high levels of LINE-1 ORF1 (Figure 2A(A-1)) and ORF2 mRNAs (Figure 2A(A-2)), as well as increased levels of exosomal miRNAs compared to PBS-treated cells, or exosomes from OH controls. The levels of ICAM1 (Figure 2B(B-1)), AGL (Figure 2C(C-1)), RGS3 (Figure 2F(F-1)), RGS13 (Figure 2G(G-1)), VCAM1 (Figure 2I(I-1)) and TGFβ1 (Figure 2J(J-1)) mRNAs were significantly upregulated in recipient cells. In contrast, PKIA (Figure 2D(D-1)), RPM39 (Figure 2E(E-1)), and VAMP3 (Figure 2H(H-1)) were either unchanged or slightly diminished following treatment with L-CAN exosomes. In sharp contrast, exosomes from OH controls and E-CAN markedly inhibited LINE-1 mRNAs and the relative expression of all genes within the LINE-1 genetic regulatory network (Figure 3A–J). Collectively, these data show that the miRNA cargo of exosomes impacts regulatory control of the LINE-1 machinery in human bronchial epithelial cells, with late-stage NSCLC derived exosomes activating miRNA-regulated oncogenic signaling in naïve recipient cells.

## 3. Discussion

miRNAs have gained considerable attention among the various exosome cargos due to their involvement in signaling pathways that contribute to NSCLC proliferation, invasion, and metastatic progression [20,21,22]. This is best exemplified by a recent demonstration that the release of exosome-derived miRNAs to local and distant areas is involved in the formation of tumor niches during metastasis [7]. Therefore, crucial steps toward future development of clinically relevant interventions for the treatment of NSCLC should focus on the identification of exosomal miRNAs and their roles in oncogenesis. In previous studies we identified a panel of eight exosomal miRNAs in plasma-derived exosomes from patients with NSCLC [23]. In the present study, we extend these findings to investigate the impact of NSCLC-associated miRNAs on oncogenic signaling in naïve lung bronchial epithelial cells, with a focus on genes within the LINE-1 regulatory network identified as putative targets including, ICAM-1, AGL, VCAM1, VAMP3, RBM39, PKIA, and RGS.

Biological discretization of the LINE-1 genetic regulatory network identified adhesion, inflammation, and cellular metabolism as key processes leading to disruption of epithelial-to-mesenchymal (EMT) programming [24,25]. One of the most significant findings of the present study was the functional linkage between ICAM-1 and miR-21-5p, miR-146a-5p, miR-221-3p, and miR-222-3p. ICAM1 is an immunoglobulin implicated in metastasis via homophilic interactions that enhance circulating tumor cell cluster formation and tumor-endothelial cell adhesion and migration [26]. Exosome-derived miR-21 upregulates VEGF and promotes malignant transformation of human bronchial epithelium [27]. miR-21 also stimulates Toll-like receptors TLR7 and TLR8 in immune cells to promote tumor growth and metastasis [28]. Another exosomal miRNA, miR-146a-5p, has been linked to NSCLC cell survival and migration by directly inhibiting the inhibitory functions of TRAF6 on cancer cell proliferation, migration, and resistance to apoptosis [29]. miR-221/222 regulate apoptosis and NSCLC tumorigenesis by targeting apoptotic peptidase activating factor 1 [30]. Furthermore, miR-221/222 interact with the tumor suppressors PTEN and TIMP3 to regulate TNF-regulated apoptosis, inflammation, and tumorigenesis. Thus, multiple microRNAs present in the exosomes of NSCLC patients regulate key inflammatory, proliferative, and apoptotic pathways through modulation of ICAM-1 function.

AGL was identified as a target of let-7b-5p, with loss of AGL expression associated with FAK activation and acquisition of anchorage-independent growth of NSCLC cells [31]. Higher levels of Let-7b-5p were observed in late-stage patients compared to early stages, suggesting that this miRNA may serve as a biomarker of disease progression. miR-210-3p levels inversely correlated with the expression of PKIA, a protein implicated in G-protein coupled Gs-cAMP signaling and deregulation of tumor growth [32]. miR-210-3p regulates STAT3 signaling in lung cancer to mediate EMT and metastasis [33]. Interestingly, the RBM39 gene was negatively correlated with miR-221-3p, with low levels of endogenous RBM39 associated with marked increases in cell proliferation and migration [34]. miR-126 exerts dual regulatory effects on angiogenesis, with either positive or negative regulation of endothelial progenitor cells and angiogenesis as a function of cell type and strand-specific functions [35,36]. RGS3 inhibits the activity of miR-126-3p [37] and neutralizes the antiproliferative effects of TGF-β in cancer cells [38].

Let-7b-5p targets VAMP3, while miR-126-3p targets VCAM1. VAMP3 is involved in the regulation of integrins and cellular trafficking and migration [39,40,41]. Of interest is the role of VAMP3 in delivery of microvesicular cargos through interactions with tetraspanin CD9 and regulation of MT1-MMP distribution in microvesicles [42]. The CEBPD transcription factor enhances cancer resistance through VAMP3-mediated autophagy activation, increasing PD-L1 levels and suppressing CD8+ T-cell-mediated immune response [43]. A crucial member of the immunoglobulin superfamily, VCAM1 triggers EMT and promotes adherence to the endothelium and formation of pseudopodia and invadopodia [44]. TGFβ1 has been identified as a confirmed target of both miR-21-5p and miR-146a-5p [45], with overexpression of oncogenic miR-21 in NSCLC cells involving decreased TGFβ1 and aberrant cell proliferation [46].

Human cells direct the loading of exosomes to selectively sort molecules into populations of varying molecular enrichment [47]. These exosomes, in turn, can transfer their contents to recipient cells to modulate their function [37,48], as shown previously with exosomal miR-146a, which mediates target gene repression and reprogramming of the cellular response to endotoxin after being taken up by recipient dendritic cells [49]. Also, exosomal miR-21 regulates the invasion and metastasis of tumors by targeting multiple tumor/metastasis suppressor genes in recipient cells [50], while miR-221/222 promotes angiogenesis in recipient endothelial cells by downregulating c-KIT, p27, and TIMP3 [51,52]. The selective loading of exosomes and transfer of exosomal contents is consistent with our observation that L-CAN exosomes activated genes within the oncogenic LINE-1 regulatory network in recipient cells, while E-CAN and control OH exosomes elicited a marked LINE-1 inhibitory response. Thus, exosomal loading and transfer likely play significant roles in NSCLC and provide new venues for therapeutic intervention, especially during the later stages of malignancy. Our findings are consistent with previous work establishing the involvement of exosomal miRNAs in angiogenesis and carcinogenesis of the lung [27,53,54].

## 4. Material and Methods

### 4.1. Identification of miRNA-Regulated Targets within the LINE-1 Genetic Regulatory Network

Putative targeting of genes within the LINE-1 regulatory network by exosomal miRNAs was evaluated using the miRNet 2.0 web-based platform [55]. Pearson correlations between miRNAs and their genetic targets in tumor and normal tissues of TCGA projects were retrieved from the CancerMIRNome online database of miRNome profiles of human cancer (http://bioinfo.jialab-ucr.org/CancerMIRNome/ accessed on 5 September 2023).

### 4.2. Exosome Isolation and Characterization

Plasma samples from ostensibly healthy (OH) individuals, early-stage NSCLC (E-CAN), and late-stage NSCLC (L-CAN) patients with similar demographic characteristics were acquired from Precision for Medicine (Norton, MA, USA). The commercial provider obtained all necessary consents prior to the collection, de-identification, and distribution of the samples. Exosomes were isolated using the exoEasy Maxi Kit (Qiagen, MD, USA; catalog number: 76064). Briefly, plasma samples were centrifuged at 16,000× *g* for 10 min to exclude remaining cells, debris, apoptotic bodies, and nuclei, and the resulting supernatant was collected for extraction of exosomes. Physical characterization of exosomes was conducted using the Nanosight instrument (NTA), followed by Western blotting to identify exosomal protein markers (Alix, Flotillin-1, and CD-9).

### 4.3. Cell Culture

The non-tumorigenic human bronchial epithelial cell line BEAS-2B was purchased from ATCC (Manassas, VA, USA) and cultured in LHC-9 Medium (1×) (ThermoFisher Scientific, Waltham, MA, USA). Cells were seeded on 6-well plates and maintained in culture media containing 10% fetal bovine serum, 100 units/mL penicillin, and 100 μg/mL streptomycin. Cultures were maintained at 37 °C in a 5% CO_2_ and 95% air environment and used after reaching confluence. All cell line batches used in the study were confirmed to be mycoplasma-free using the MycoAlert Mycoplasma Detection Kit (Lonza, Walkersville, MD, USA, Catalog: LT07-318).

### 4.4. Co-Culture Experiments

Plasma exosomes from OH controls, E-CAN, or L-CAN were added to BEAS-2B cultures for 72 h at an estimated ratio of 20:1. Sterile phosphate-buffer saline (PBS) was used as a vehicle control.

### 4.5. RNA Isolation, cDNA Synthesis and Realtime-PCR

Total RNA was extracted using the TRIzol Reagent (ThermoFisher Scientific, Waltham, MA, USA) following standard procedures. Total RNA was rinsed with 1 mL of precooled 70% ethanol, centrifuged at 12,000× *g* for 5 min at 4 °C, and dissolved in DEPC-treated water. The Reverse Transcription System (Promega, Madison, WI, USA) was used for cDNA synthesis. Briefly, the samples were incubated at 42 °C for 15 min. followed by heating at 95 °C for 5 min, and incubation at 4 °C for 5 min. Real-time PCR was performed for LINE-1 ORF1 and ORF2 mRNAs, as well as target genes within the LINE-1 regulatory network (ICAM1, AGL, PKIA, RBM39, RGS3, RGS13, VAMP3, VCAM1, and TGFβ1). We also measured the cellular levels of miR-21-5p, miR-126-3p, miR-210-3p, miR-221-3p, Let-7b-5p, miR-146a-5p, and miR-222-3p using CFX96 Touch Real-Time PCR Detection System (Biorad, Hercules, CA, USA). The PCR conditions for ICAM1, AGL, PKIA, RBM39, VAMP3, and VCAM1, along with all the examined miRNAs, were as follows: 95 °C for 10 min, followed by 40 cycles of 95 °C for 15 s and 60 °C for 1 min. The PCR conditions for RGS3 and RGS13 also included an initial denaturation of 6 min at 94 °C followed by amplification for 40 cycles, denaturation at 94 °C for 1 min, annealing at 52 °C for 1 min, and extension at 72 °C for 1.5 min. For TGFβ1, the PCR conditions included an initial denaturation at 94 °C for 2 min followed by 34 cycles of denaturation at 94 °C for 15 s, annealing at 58 °C for 30 s, elongation at 72 °C for 1 min, and a final elongation at 72 °C for 7 min. Each reaction was carried out three times and the calculation of fold changes performed using the 2^∆∆Ct^ technique, where Ct is the threshold cycle.

### 4.6. Statistical Analyses

Statistical analyses were performed using GraphPad Prism, version 9.5.0 (GraphPad Software, San Diego, CA, USA). The ANOVA and Tukey’s HSD test were employed to evaluate differences among groups. A significance threshold of *p* < 0.05 established statistical significance and *p* < 0.01 high statistical significance.

## 5. Conclusions

Collectively, our findings add to the current knowledge base by showing that late-stage NSCLC exosomes transfer their miRNA cargos to naïve lung bronchial epithelial cells where they hijack the cellular machinery to disrupt control of key cellular functions and alter genes within the oncogenic LINE-1 regulatory network. Thus, cancer-derived exosomal miRNAs may contribute to the progression of NSCLC and play important roles during the later stages of malignant progression. Functional assays to assess the phenotypic and signaling consequences of miRNA-mediated modulation of LINE-1 and its oncogenic targets will be needed to define the cellular consequences of exosomal transfer. These experiments can provide mechanistic insights into how exosomal miRNAs influence NSCLC progression and metastasis.

## Data Availability

The data presented in this study are available on request from the corresponding author.

## Supplementary Materials

The following supporting information can be downloaded at: https://www.mdpi.com/article/10.3390/ijms251910674/s1.
