# Supplementary material for: Modulation of the Oncogenic LINE-1 Regulatory Network in Non-Small Cell Lung Cancer by Exosomal miRNAs
Source: Int J Mol Sci. 2024 Oct 3;25(19):10674. doi: 10.3390/ijms251910674 (PMC11477113; doi:10.3390/ijms251910674)

Supplementary Figures:

Supplementary Figure 1: Concentration and diameter of exosomes, A-1) Control group, A-2) Early-stage NSCLC patients' group, A-3) Late-stage NSCLC patients' group. B) Western blot analyses for Alix, Flotillin-1, CD9, and Calnexin.

Supplementary Figure 1:

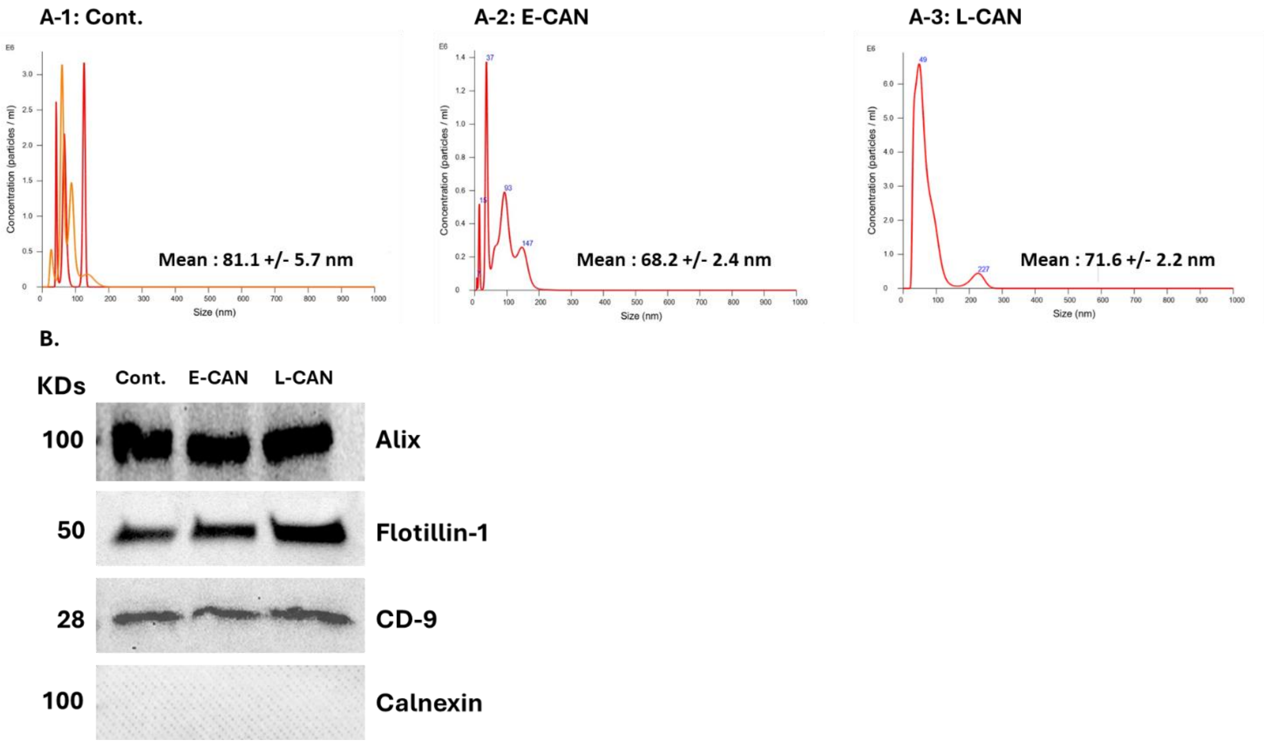

Supplement: Supplementary file 1 [file ijms-25-10674-s001.zip › ijms-3233967-supplementary.pdf]
